# Splenic red pulp macrophages are intrinsically superparamagnetic and contaminate magnetic cell isolates

**DOI:** 10.1038/srep12940

**Published:** 2015-08-11

**Authors:** Lars Franken, Marika Klein, Marina Spasova, Anna Elsukova, Ulf Wiedwald, Meike Welz, Percy Knolle, Michael Farle, Andreas Limmer, Christian Kurts

**Affiliations:** 1 Institute of Experimental Immunology, Rheinische Friedrich-Wilhelms-University, Sigmund-Freud-Straße 25, 53105 Bonn, Germany; 2 Faculty for Physics and Center for Nanointegration Duisburg-Essen (CENIDE), University Duisburg-Essen, Lotharstraße 1, 47057 Duisburg, Germany; 3 Institute of Molecular Immunology, Technical University Munich, Schneckenburgerstr. 8, 81675 Munich, Germany; 4 Clinic for Orthopaedics and Trauma Surgery Bonn, University Clinic Bonn, Sigmund-Freud-Straße 25, 53105 Bonn, Germany

## Abstract

A main function of splenic red pulp macrophages is the degradation of damaged or aged erythrocytes. Here we show that these macrophages accumulate ferrimagnetic iron oxides that render them intrinsically superparamagnetic. Consequently, these cells routinely contaminate splenic cell isolates obtained with the use of MCS, a technique that has been widely used in immunological research for decades. These contaminations can profoundly alter experimental results. In mice deficient for the transcription factor SpiC, which lack red pulp macrophages, liver Kupffer cells take over the task of erythrocyte degradation and become superparamagnetic. We describe a simple additional magnetic separation step that avoids this problem and substantially improves purity of magnetic cell isolates from the spleen.

Magnetic cell separation (MCS) is a technique to isolate specific cell subsets from any desired source with relatively little effort and high purity. This technique is convenient, fast and yields large numbers of cells and is therefore employed in many research and clinical institutions worldwide^1^,^2^. A cell subset to be isolated from a cell suspension is targeted with specific antibodies linked to magnetic nanoparticles. These are then passed over a separation column under the influence of a strong magnetic field. Cells carrying the magnetic nanoparticles bind to the column and can be eluted after switching off the magnetic field. MCS is particularly useful for immunological studies, where various immune cell subsets need to be isolated for subsequent analysis like adoptive transfer experiments, RT-PCR analysis or to study their cytokine secretion by ELISA. In comparison to flow cytometric cell sorting, purity of cell isolates is usually lower and less parameters can be used. On the other hand, MCS is time-saving, yields higher cell numbers and does not require expensive machinery and specially trained personnel. Therefore, this technique is widely employed in immunology.

The spleen is a particularly convenient source for murine immune cells: It contains many T and B cells, as well as several large subsets of CD11c^+^ dendritic cells (DCs) and macrophages^3^. When large numbers of such immune cells are needed, they are commonly isolated from the spleen by MCS for purposes like studying gene expression or cellular functions. Purities higher than 95% are difficult to achieve by MCS, but some assays may be critically affected even by smaller contaminations, for example measuring mRNA expression by RT-PCR.

In this study, we describe that MCS isolates from the spleen, and under certain conditions from the liver, are routinely affected by distinct macrophage contaminants and that these may profoundly affect experimental results. We also describe how to avoid this problem.

## Results

### RPM contaminate MCSs from the murine spleen

**Figure 1:**
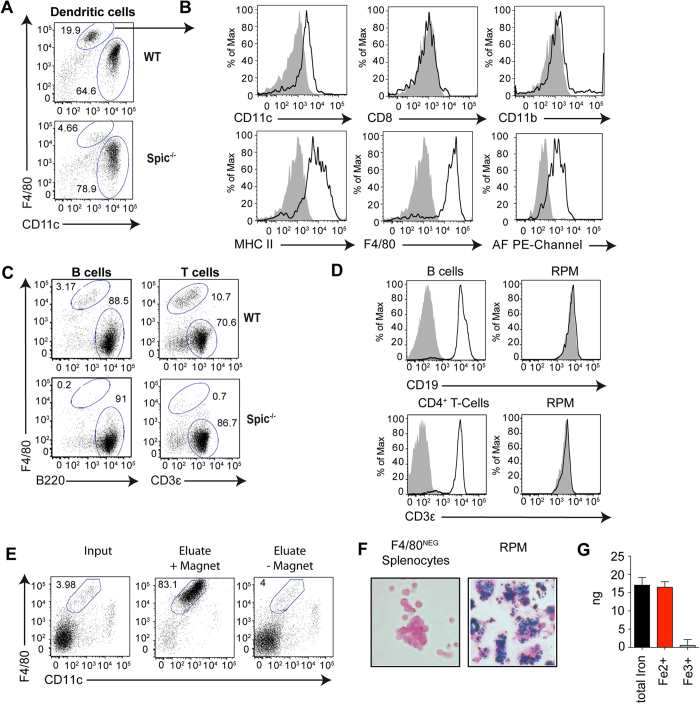
RPM contaminate MCSs. (**A**) Cells isolated by MCS using CD11c-specific nanoparticles from spleens of Spi-C-deficient and –competent mice, stained for F4/80 and CD11c. (**B)** Expression of surface markers on RPM identified as shown in (a). Isotype controls given as gray background and autofluorescence (AF) in the 488 nm channel by analyzing unstained cells. (**C**) RPM contaminations in B and T cells isolated with CD19- or CD3ε-specific mircobeads. (**D**) Expression of CD19 on B-Cells and RPM (left), as well as expression of CD3ε on CD4 + T cells and RPM (right) in the 650 nm channel. RPM were identified as shown in (a), B cells as B220^+^ CD11c^−^, CD4^+^ T cells as CD4^+^ CD11c^−^. Isotype controls given as gray background. (**E**) RPM enrichment in splenocytes passed over MCS columns (input) without using paramagnetic nanoparticles, either applying a magnetic field (+Magnet) or not (–Magnet). (**F**) Ferric iron content of F4/80-negative splenocytes and RPM detected with Prussian blue staining. (**G**) Colorimetric determination of the iron content of RPM. Depicted is the total iron amount isolated from 1 × 10^6^ RPM according to the manufactures protocol. Results are shown for one representative of two to three individual experiments using 2–4 mice per group. Error bars, s.d. (n = 2–4 mice); *p < 0.05; ***p < 0.001.

When we isolated CD11c^+^ dendritic cells (DCs) from this organ with the use of MCS, we noted that in addition to the two classical splenic DC subsets expressing either the marker CD8 or CD11b^4^,^5^, 20–30% of the cell isolate constituted a third subset (Fig. 1A, upper dot-plot). These cells showed high autofluorescence at various visible light frequencies and displayed the surface phenotype CD11c^lo^ F4/80^+^ MHC II^+^ CD8^–^ CD11b^–^ (Fig. 1B, Supplementary Figure 1), which is characteristic of red pulp macrophages (RPM)^6^. Confirming this classification, these cells were scarce in isolates from mice lacking the transcription factor SpiC (Fig. 1A, lower dot-plot), which is required for RPM development^6^.

RPM express low levels of CD11c, which may explain their presence in splenic CD11c^+^ DC isolates. However, RPM were present also when we isolated B cells or T cells using nanoparticles targeting CD19 and the CD3ε, respectively (Fig. 1C), although these markers are not expressed by RPM (Fig. 1D). Their frequency was 5–10%, presumably because CD3^+^ T or CD19^+^ B cells are more abundant in the spleen than DCs, diluting out the RPM. Even more surprisingly, RPM were detected also when no nanoparticle-bearing antibodies were used (Fig. 1E), indicating that RPM intrinsically bind to the magnetic columns. When the magnetic field was removed, RPM did not bind to the columns any more (Fig. 1E), hinting at para- or superparamagnetic properties of RPM.

### RPM are superparamagnetic

**Figure 2:**
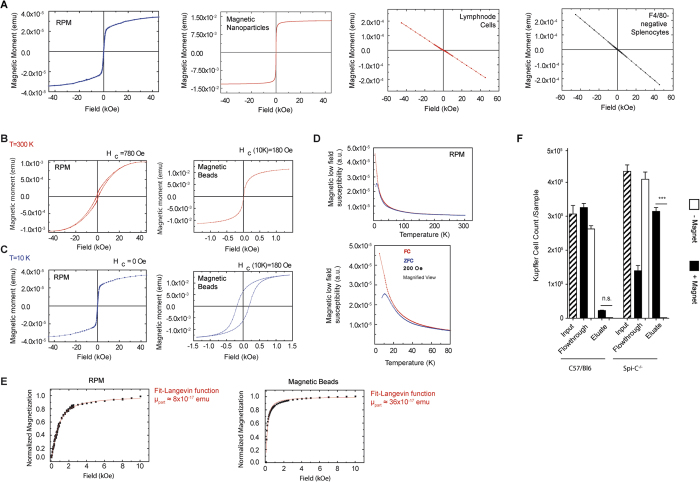
RPM show superparamagnetic properties comparable to those of commercially available magnetic beads. (**A**) SQUID analysis of magnetic properties of RPM, F4/80^−^splenocytes, lymph node cells and paramagnetic nanoparticles as a positive control. Depicted is the magnetic moment at room temperature as a function of the external magnetic field applied. Characteristic magnetization curves for superparamagnetic (RPM, Magnetic Nanoparticles) and diamagnetic behavior (lymph node cells, F4/80-negative Splenocytes) were recorded. (**B**) Ferromagnetic response of RPM (left graph) and magnetic nanoparticles (right graph) at 10 K measured in a SQUID magnetometer. (**C**) Superparamagnetic response of RPM (left graph) and magnetic nanoparticles (right graph) at 300 K measured in a SQUID magnetometer. (**D**) Temperature dependence of low-field susceptibility of RPM was measured in a magnetic field of 200 Oe after the sample was cooled in zero magnetic field (ZFC, blue) and in an external magnetic field of 200 Oe (FC, red). (**E**) Magnetic moment of one superparamagnetic (likely Fe Oxide) nanoparticle in the RPM or one magnetic nanoparticles obtained from a fit of superparamagnetic M(H, 300K) dependencies. (**F**) Enrichment of liver Kupffer cells in SpiC-deficient and—competent mice after passage over MCS columns (input) without using paramagnetic nanoparticles, either applying a magnetic field (+Magnet) or not (–Magnet). Results are shown for one representative of two to three individual experiments using 2–4 mice per group. Error bars, s.d. (n = 2–4 mice); *p < 0.05; ***p < 0.001.

A main function of RPM is to degrade aged or damaged erythrocytes. Consequently, RPM accumulate heme degradation products that render them autofluorescent^7^,^8^ and iron, which can be visualized by Prussian blue staining (Fig. 1F) and by colorimetry (Fig. 1G). We hypothesized that iron accumulation might be the reason for the magnetic properties of RPM and examined these cells with a superconducting quantum interference device (SQUID), a very sensitive magnetometer. This analysis confirmed strong superparamagnetic properties in RPM at room temperature, comparable to those in commercially available magnetic nanoparticles that served as a positive control, but not in splenocytes depleted of RPM or in lymph node cells lacking RPM (Fig. 2A). At a temperature of 10 K RPM showed a ferromagnetic response (open hysteresis with a coercive field of 780 Oe) (Fig. 2B,C), while magnetic nanoparticles showed a ferromagnetic hysteresis M(H) loop at 10 K with a coercivity of 180 Oe (much lower in comparison to 780 Oe measured at 10 K for RPM) (Fig. 2B,C). By analysis of the temperature dependence of low-field susceptibility of RPM, an effective magnetic anisotropy constant (K_eff_ ≈ 5.8 × 10^5^ erg/cm^3^) was determined, which is consistent with ferrimagnetic iron oxides^9^. Additionally, an irreversibility in the zero magnetic field curves was observed up to 75 K, indicating the presence of ferromagnetic particles in RPM (Fig. 2D). A fit of superparamagnetic M(H, 300 K) dependencies revealed that the magnetic moment pro magnetic cell particle at 300 K was comparable to that of the reference magnetic nanoparticles (Fig. 2E). Thus, RPM contain iron oxide particles that render them superparamagnetic, similar to commercially available paramagnetic nanoparticles.

### Kupffer cells also show superparamagnetic behavior

Kupffer cells, a hepatic macrophage type, contribute to the phagocytosis of erythrocytes in certain diseases like sickle cell anemia or malaria^10^,^11^. We therefore asked whether also these cells accumulate iron and contaminate magnetic liver cell purificates due to superparamagnetism. When we passed liver single cell suspensions over magnetic columns, Kupffer cells were not significantly enriched after passage of the columns (Fig. 2F). However, when we used liver single cell suspensions from SpiC-deficient mice, Kupffer cells were enriched 10fold in eluates and reduced by 66% in the flow-through (Fig. 2F). This indicates that in situations when RPM are absent, for example after splenectomy, Kupffer cells take over their role in erythrocyte degradation, become superparamagnetic and may then contaminate magnetic liver cell isolates.

### A technique to remove RPM from splenic cell isolates

**Figure 3:**
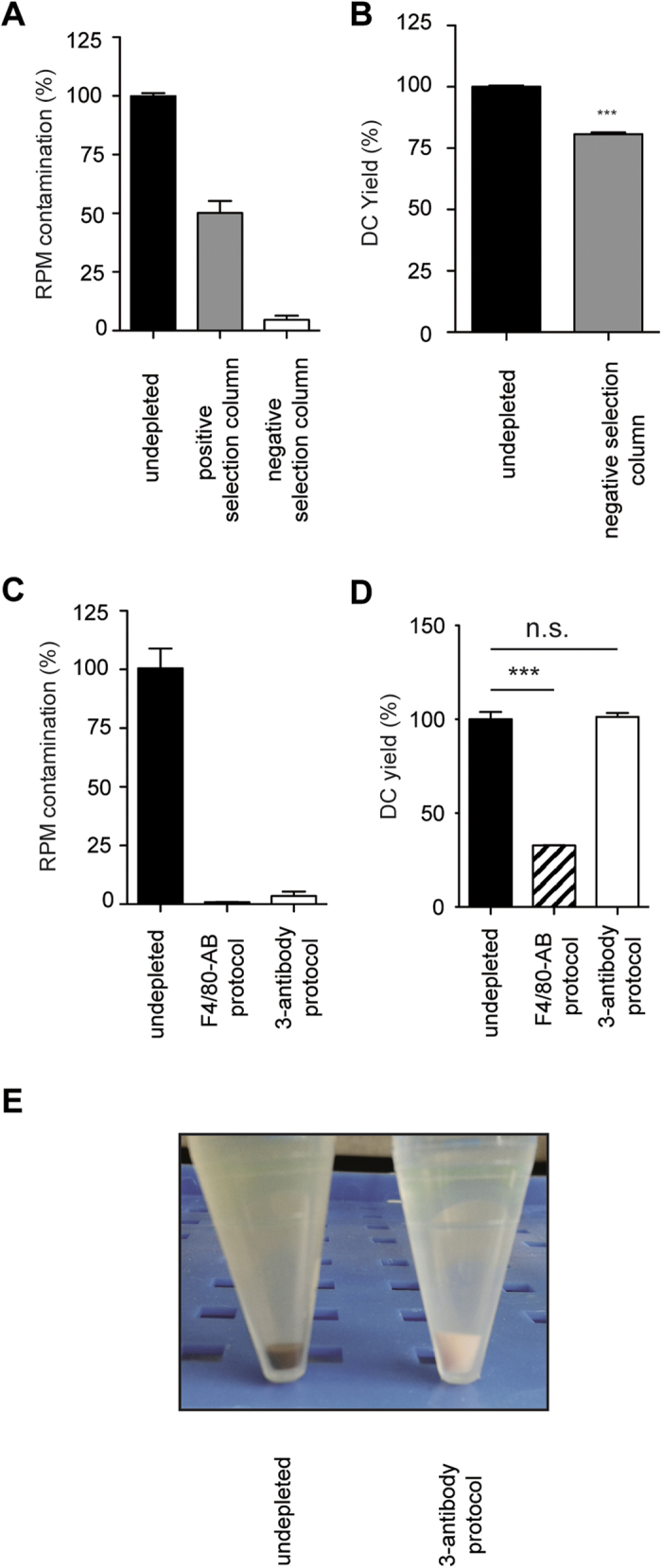
Removing RPM by MCS. (**A**) RPM contaminations in CD11c^+^ cell isolates obtained without RPM-depletion or after RPM-depletion on the indicated column. (**B**) Relative yield of splenic DCs after RPM-depletion using the negative selection column compared to isolation without depletion. (**C**) RPM contaminations in CD11c^+^ cell isolates obtained without RPM-depletion or after RPM-depletion using the indicated protocol. (**D**) Relative yield of splenic DCs after RPM-depletion using the two antibody-based depletion protocols compared to isolation without depletion. (**D**) RPM contaminations in B or T cell isolates with or without RPM-depletion using the F4/80-bead protocol. (**E**) CD11c^+^ cell isolates obtained without (left tube) or with RPM depletion using the 3-antibody protocol (right tube). Results are shown for one representative of two to three individual experiments using 2–4 mice per group. Error bars, s.d. (n = 2–4 mice); *p < 0.05; ***p < 0.001.

We next developed techniques to remove RPM contaminations from splenocyte suspensions. Passing splenocytes over commercial positive selection columns removed only 50% of the RPM, whereas a negative selection column, which features a slower flow rate, removed 95%, albeit at the expense of a 20% lower DC yield (Fig. 3A,B). A similar removal efficiency was achieved by targeting RPM with F4/80-specific antibodies and passing them over a positive selection column (Fig. 3C). While this was suitable for lymphocyte isolation, it removed about 60% of the CD11b^+^ splenic DCs (Fig. 3D), which express low F4/80 levels^12^. Selective removal of > 99% of the RPM was achieved using a cocktail of antibodies against FPR-1, CCR3 and PILRα (Fig. 3C). RPM-depletion was evident to the naked eye: Splenocytes containing RPM displayed the familiar rusty brown color of iron oxide, while RPM-depleted splenocytes appeared white (Fig. 3E). Subsequent isolation of CD11c^+^ cells from RPM-depleted splenocytes returned the same number of DCs compared to undepleted splenocytes (Fig. 3D), indicating that the 3-antibody RPM depletion protocol did not noticeably compromise the DC yield. As PILRα is expressed also on neutrophils and CCR3 on eosinophils (data not shown), it may be impossible to adapt this DC isolation protocol to granulocytes.

### Functional consequences of RPM contaminations

**Figure 4:**
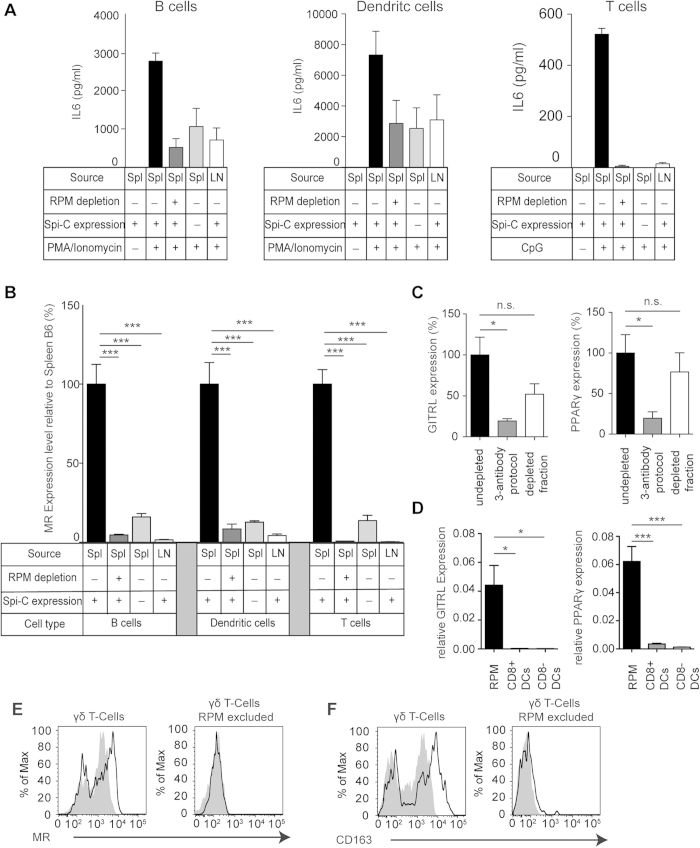
Functional consequences of RPM contaminations. (**A**) IL-6 concentrations in supernatants from B cell (left graph), DC (middle graph) or T cell (right graph) cultures obtained by MCS from spleens or the mesenteric lymph nodes with or without RPM-depletion using the 3 antibody-based protocol. (**B**) mRNA levels of MR determined by RT-PCR. Cell type, source tissue, SpiC-expression and RPM-depletion as indicated. (**C**) mRNA-expression of GITRL (left graph) or PPARγ (right graph) in CD11c^+^ cell isolates with or without RPM-depletion using the 3 antibody-based protocol. (**D**) mRNA-expression of GITRL (left graph) or PPARγ (right graph) in sorted RPM, CD11c^+^ CD8^+^ DC or CD11c^+^ CD8^−^ DCs. (**E**, **F**) γδ T cells stained for the RPM-Marker MR (**E**, black line) or CD163 (**F**, black line) or the corresponding isotype (grey background). Left histograms: marker expression without excluding RPM, right histograms: marker expression when the analysis gate is free of autofluorescent RPM. The average (mean) values ± s.e.m. are shown. Results are shown for one representative of two to three individual experiments using 2–4 mice per group. Error bars, s.d. (n = 2–4 mice); *p < 0.05; ***p < 0.001.

MCS has been extensively used for decades by immunologists to conveniently isolate subsets from splenocyte suspensions at large numbers^1^,^13^,^14^,^15^. Therefore, we next examined whether RPM contaminations have functional consequences and whether these can be avoided by our depletion protocol. To this end, we isolated B cells, DCs and T cells from spleens by MCS and stimulated them with PMA/ionomycin. The resulting supernatant contained IL-6 (Fig. 4A) and TNF-alpha (Supplementary Figure 2A), both typical macrophage cytokines. Much lower concentrations were detected when B cells or DCs were isolated from sources lacking RPM, such as spleens of SpiC KO mice or lymph nodes from wild-type mice (Fig. 4A, Supplementary Figure 2A), suggesting that IL-6 and TNF-alpha had been primarily produced by contaminating RPM. Indeed, splenic B cells or DCs isolated with the use of our by our RPM depletion protocol produced very little IL-6 or TNF-alpha (Fig. 4A, Supplementary Figure 2A). We obtained similar results when we used T cells isolated by MCS (Fig. 4A, Supplementary Figure 2A). No reduction of the chemokines CCL3 and CCL4 after RPM-depletion was observed (Supplementary Figure 2BM), confirming that our RPM-depletion protocol does not reduce cytokine/chemokine levels per se.

We also asked whether RPM contaminations affect antigen presentation assays. However, when isolated RPM very poorly presented the model antigen OVA to OVA-specific CD4^+^ T cells (Supplementary Figure 3), consistent with a previous report^16^.

To provide another example for such influences, we performed RT-PCR on splenic T cells, B cells and DCs isolated by MCS. mRNA encoding the macrophage markers CD206 (mannose receptor, MR) and CD163 was detected in all these isolates (Fig. 4B, Supplementary Figure 4). However, these markers were absent when we removed RPM from splenocytes by our depletion method or when we used sources lacking RPM (Fig. 4B, Supplementary Figure 4), whereas typical DC, T and B cell markers like CD8β, CD3ε, CD11c, DEC205, CD19 or B220 were not reduced (Supplementary Figure 5). Similar conclusions were drawn when we determined expression levels of genes associated with iron metabolism that are active in RPM, namely Hmox1, Fpn1 and VCAM-1: These genes were present in DC isolates from the spleen unless RPM were depleted (Supplementary Figure 6). This indicated that RPM contaminations influence RT-PCR analysis of splenocyte subsets isolated by MCS. This may explain previous reports describing mRNA expression of the immunoregulatory molecules GITRL (*TNFSF18*) and PPARγ (*NR1C3*) in splenic DCs^17^,^18^. We confirmed this in DCs purified by MCS (Fig. 4C) However, these molecules were absent from splenocytes depleted of RPM, from splenic DCs purified by cell sorting, but present in sorted RPM (Fig. 4D), indicating that they were in fact expressed by contaminating RPM. Thus, RPM contaminations can profoundly influence functional analysis of splenocyte isolates.

### The autofluorescence of RPM may affect fluorescence-activated cell sorting

An alternative method for immune cell isolation is fluorescence-activated cell sorting (FACS), which is considered more specific than MCS. Although FACS is unaffected by the superparamagnetism of RPM, it is hampered by their strong autofluorescence^19^, which may feign binding of fluorescent antibodies, so that RPM appear in the sorted cell population although they do not fulfill the sorting criteria. This may explain why γδ T cells were reported to express the mannose receptor and CD163 in a recent transcriptional analysis, which used an analysis gate that overlapped with the region of autofluorescent cells (http://www.immgen.org/databrowser/viewPopulationPDF?popId=357). We failed to detect these macrophage molecules in γδ T cells after excluding RPM (Fig. 4E,F).

## Discussion

We here report that erythrocyte-degrading cells, especially RPM and in their absence Kupffer cells, are superparamagnetic and contaminate cell isolation by MCS. If unnoticed, these contaminations can profoundly affect immunological readouts, so that it is difficult to distinguish between properties of macrophages and the cells of interest. The contamination of MCS isolates of CD11c^+^ DCs by RPM is not surprising given that RPM express low levels of this molecule. We found that up to one third of a CD11c^+^ splenocyte subset might represent RPM rather than DCs depending on the experimental conditions used. We conclude that CD11c^+^ cells obtained by MCS are a mixture of RPM and DCs. One reason why this problem may escape attention is the high autofluorescence of RPM. Although it is common knowledge that the spleen contains numerous autofluorescent cells, it is less generally known that these cells are RPM^6^. Thus, RPM phenotypically resemble dead cells, which are often autofluorescent either^20^,^21^. Consequently, RPM may be excluded from flow cytometric analysis when dead cell exclusion gates are applied, and become invisible during standard flow cytometric analysis. Nevertheless, they are still present in the isolates and may affect analysis by RT-PCR or cell culture assays.

Surprisingly, RPM contaminations were seen also when we isolated cells using markers that RPM do not express, such as B and T cells. As these cells are more frequent in the spleen than DCs, the proportion of RPM contaminations was lower compared to those in DC isolates. We noted that RPM were also enriched when no magnetic nanoparticles were used at all, but this was not the case when the magnetic field was absent. This indicated that RPM do not bind non-specifically to the separation column but are rather retained due to intrinsic magnetic properties. As RPM have a prominent role in erythrocyte degradation^3^,^22^, we reasoned that the concomitant iron uptake might render them intrinsically magnetic. RPM indeed contained massive amounts of ferric iron and showed superparamagnetic properties at room temperature as reveals by a SQUID magnetometer. The effective magnetic anisotropy constant calculated for RPM was compatible with that of ferrimagnetic iron oxides^9^. The most plausible conclusion is that RPM contain small, ferrimagnetic iron oxide nanoparticles, which under normal circumstances are randomly oriented within the cell. Exposure of the RPM to a strong magnetic field aligns the nanoparticles and magnetizes the cells. The exact molecular makeup of these iron clusters however is still unclear and needs to be studied further. Most likely the iron is part of hemosiderin complexes, which has been reported to exhibit superparamagnetic properties^23^. These superparamagnetic properties may possess further functional consequences beyond those described here, for example unexpected immunological reactions under the influence of a strong external magnetic field.

We then demonstrated that the contamination of cell isolates by RPM can have functional consequences, which needs to be taken into account when interpreting data from experiments involving MCS from the murine spleen and liver: For example, after stimulation splenic B and T cells seemed to produce typical macrophage cytokines such as IL-6 and TNF, but these were in fact produced by contaminating RPM. Also IL-6 and TNF-production by splenic DCs was much lower when RPM contaminations were removed. It is possible that RPM contaminations might have influenced previous reports that splenic B cells produced IL6 when stimulated with CpG oligonucleotides^24^.

RPM contaminations also perturb gene expression analysis by RT-PCR, another common technique in immunology. In cell isolates of B cells, T cells- and DCs obtained by MCS we detected expression of the typical RPM-molecules MR and CD163. These were not present after RPM had been depleted or when cells had been isolated from a source lacking RPM, such as lymph nodes or the spleens of SpiC deficient mice. Thus, the expression of any gene active in RPM might be attributed to other cells if RPM are not removed from the isolate. There are examples in the literature were this may have happened^17^,^18^.

FACS is often considered to yield higher cell purity than MCS. However, it requires expensive machinery and is more time-consuming and expensive per cellular yield, and thus less convenient for routine applications on a daily basis. Furthermore, there is evidence that the autofluorescence of RPM affects also FACS. The reason for such autofluorescence is most likely the acquisition and degradation of heme metabolites during erythrocyte metabolism^19^. Thus, the autofluorescence of RPM can have two adverse effects during cell sorting: first, it can lead to their exclusion from flow cytometric analysis when dead cell gates are applied, and second it can lead to their inclusion into flow cytometric sorting gates when dead cell gates are not stringent enough.

In conclusion, RPM are superparamagnetic and contaminate cell purifications by MCS. These contaminations may affect immunological readouts, so that macrophages functions may be falsely attributed to other splenic cell types. The additional MCS step described here avoids these contaminations, substantially increases the purity of cell isolates and avoids potential alterations of experimental results.

## Material and Methods

### Reagents and mice

All reagents were from Sigma-Aldrich unless specified otherwise. All mice had been backcrossed to C57BL/6 at least ten times, were bred under specific pathogen–free conditions at the central animal facility of the University Clinic of Bonn and were used at 8–12 weeks of age. Animals were maintained with approval of the Bezirksregierung Köln of the German state of North Rhine-Westphalia. All methods were carried out in accordance with the approved guidelines and regulations.

### Cell preparation

Single cell suspensions of splenocytes and lymph nodes were generated by digesting the tissue with 50 μg/ml DNAse and 400 U/ml Collagenase at 37 °C for 20 minutes and passing it through a metal sieve and a sterile 50 μm nylon mesh afterwards. For the isolation of non-parenchymal liver cells, livers were perfused *in situ* with 0.05 % Collagenase, mechanically dissociated and digested with 0,04 % Collagenase at 37 °C for 20 minutes and passed through a 250 μm cell strainer. After separation by a 25 % and 50 % Percoll gradient centrifugation for 30 min at 1350 × g at 4 °C, non-parenchymal liver cells were collected from the interface and passed through a 50 μm nylon mesh.

### Antibodies and flow cytometry

Cells were stained with the following fluorochrome-conjugated antibodies: anti-B220 (eBioscience, clone RA3-6B2), anti-CD3e (eBioscience, clone 145-2C11), anti-CD8a (BD, clone 53-6.7), anti-CD11b (eBioscience, clone M1/70), anti-CD11c (Bio Legend, clone N418), anti-CD14 (Bio Legend, clone SA14-2), anti-CD19 (eBioscience, clone eBio1D3), anti-CD64 (Bio Legend, clone X54-5/7.1), anti-CD68 (AbD Serotec, clone FA-11), anti-CD163 (Bioss, polyclonal), anti-CD115 (eBioscience, clone AF598), anti-CCR3 (Bio Legend, clone J073E5), anti-F4/80 (Bio Legend, clone CI:A3-1), anti-FPR-1 (Bioss, polyclonal), anti-MHC II (Bio Legend, clone M5/114.15.2), anti-MR (AbD Serotec, clone MR5D3) and anti-PILRa (R&D Systems, polyclonal). Fc receptors were blocked with 1.5mg/ml human IgG (Privigen). Dead cells were excluded using the Hoechst 33342 dye. Only events that appeared single in forward-scatter width were analyzed. The gating strategy is shown in Supplementary Figure 7. A FACSCanto II and FACSDiva software (BD) were used for flow cytometry and data were analyzed with FlowJo software (TreeStar).

### MACS enrichment

DCs, T Cells and B Cells were enriched from single-cell suspensions of murine splenocytes using MCS (Miltenyi) according to the manufacturers protocol. Briefly, single cell suspensions were either incubated with anti-CD11c or anti-CD19 antibody-labeled nanoparticles (DCs and B cells) or with Biotin-conjugated antibodies specific for CD3e. The latter were afterwards incubated with anti-Biotin nanoparticles (T cells). The cells were then washed with MACS buffer and separated from the non-labeled cells on LS separation columns according to the manufacturer’s protocol.

### RPM depletion

To deplete Red Pulp Macrophages (RPM) from splenic single cell suspension, 5 × 10^7^ splenocytes were incubated with 2.5 μg of the indicated biotin- or PE-conjugated antibodies and 1.5 mg/ml human IgG (Privigen) for 25 minutes at 4 °C. After a washing step the cells were incubated with either with anti-Biotin or anti-PE microbeads (Miltenyi), depending on the primary antibodies for 15 minutes at 4 °C. After another washing step the cell suspension was passed over a LS separation column, which was washed three time with 2 ml MACS-Buffer (Miltenyi). The flow through was collected and used for downstream applications.

### RPM purification

To purify RPM, single cell suspensions of splenocytes were passed over LS separation columns (Miltenyi) without further treatment. Before being removed from the magnet, the columns were washed three times with 2 ml MACS buffer (Miltenyi). After removal of the magnet, the RPM were eluted from the separation column with 5 ml MACS buffer.

### RT-PCR

Total RNA was isolated using the RNAzol RT according to the manufactures protocol (Sigma-Aldrich). cDNA synthesis was performed using the SuperScript III cDNA Synthesis Kit (Invitrogen). Real-time PCR using PerfeCTa SYBR Green FastMix was performed with the Light Cycler 480 System (Roche) with oligonucleotide primers reported in Supplementary Table 1 that were designed using NCBI primer blast software and purchased from Invitrogen. Primer specificity was confirmed by melting curve analysis. The results are expressed as fold-increase mRNA expression of the gene of interest normalized to *GAPDH* expression by the DDCt method. Reported values are the mean and s.e.m. from two independent experiments performed on different days (*n* = 3).

### Iron Detection

For staining of ferric iron, 400000 RPM or F4/80-negative splenocytes were purified as described and plated on glass coverslips. Afterwards the cells were washed two time with PBS and fixed for 10 minutes at room temperature with 4 % PFA in PBS. The fixed cells were washed two times with cold PBS and ferric iron was stained using a ferric iron stain kit (Sigma-Aldrich) according to the manufacturer’s protocol. The coverslips were mounted using Immu-Mount (Thermo) and analyzed on an IX71 fluorescent microscope (Olympus).

Qunatification of of Fe^2+^ and Fe^3+^ in isolated RPM was performed by using an Iron Colorimetric Assay Kit (Abcam) according to manufacturer’s instructions.

### Cytokine profiling

Cytokine and chemokine concentrations in cell culture supernatants from MACS-purified cells were analyzed using the FlowCytomix Multiplex Technology (Affymetrix). In brief, 200000 cells were plated in 200 μl media in 96-wells and were activated either using 50 ng/ml PMA and 500 ng/ml Ionomycin or with 10 μg/ml CpG. 18 hours later the cell culture supernatants were collected, soluble cells were removed by centrifugation and the chemokine and cytokine levels were analyzed according to the manufacturers protocol.

### SQUID

A commercial Quantum Design MPMS XL SQUID magnetometer with a sensitivity of 10^−8^ emu, a magnetc field range of −50 to 50 kOe and a temperature range of 4 to 350 K was used for the magnetic measurements. Single cell suspensions of sorted RPM or magnetic nanoparticles were dried on Si substrates and measured under He atmosphere.

### Statistics

Results are expressed as mean ± SEM. Differences between multiple groups were assessed by using an one-way ANOVA in combination with a Bonferroni multiple-comparison test, differences between two groups were assessed by using a 2-tailed paired Student’s *t* test (Prism 4, Graphpad Software). *P* values of less than 0.05 were considered significant. Tests were reported only where data met assumptions of tests. On the basis of preliminary experimental data a power analysis of 0.8 with p < 0.05 indicates a minimum number of 2 samples/purifications per group, but in some cases 3 samples/purifications per group were used.

## Additional Information

**How to cite this article**: Franken, L. *et al.* Splenic red pulp macrophages are intrinsically superparamagnetic and contaminate magnetic cell isolates. *Sci. Rep.* **5**, 12940; doi: 10.1038/srep12940 (2015).

## Supplementary Material

Supplementary Information
