# Supplementary material for: Splenic red pulp macrophages are intrinsically superparamagnetic and contaminate magnetic cell isolates
Source: Sci Rep. 2015 Aug 11;5:12940. doi: 10.1038/srep12940 (PMC4531335; doi:10.1038/srep12940)
Supplement: Supplementary Information [file srep12940-s1.pdf]

Supplementary Information

Splenic red pulp macrophages are intrinsically superparamagnetic and contaminate magnetic cell isolates

Lars Franken, Marika Klein, Marina Spasova, Anna Elsukova, Ulf Wiedwald, Meike Welz, Percy Knolle, Michael Farle, Andreas Limmer and Christian Kurts

Supplementary Figure 1:

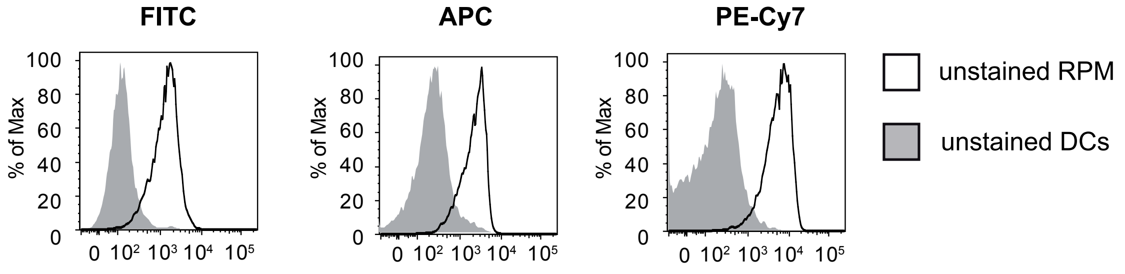

Supplementary Figure 1: Flow cytometrical analysis of the autofluorescent properties of RPM and DCs. The autofluorescence was analyzed in the indicated channel. The RPM signal is depicted as a black line, the DC signal as grey background.

Supplementary Figure 2:

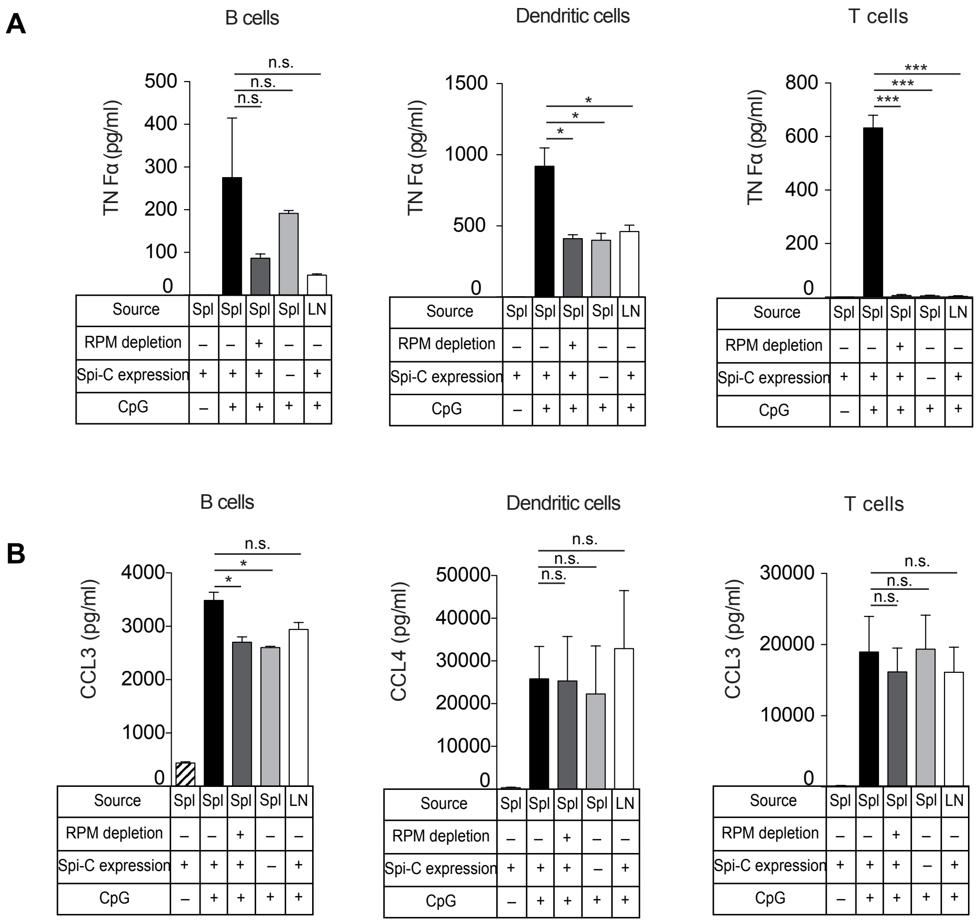

Supplementary figure 2: RPM-contaminations alter cytokine concentrations in supernatants from in vitro cultures of conventional dendritic cells, T Cells and B-Cells. B cells, T cells and DCs were obtained by magnetic cell purification from spleens or the mesenteric lymph nodes without or with RPM-depletion using the 3 antibody method. Cells were activated as described and 18 hours later supernatants were collected and cytokine levels were analyzed. (**A**) TNF-alpha concentrations in supernatants of B cells, DCs and T cell cultures. (**B**) CCL3-concentrations in supernatants of B- or T cell cultures or CCL4-concentrations in supernatants of DC cultures. Results are shown for one representative of two to three individual experiments using 3 mice per group. Error bars, s.d. (n=3 mice); *P< 0.05; ***P< 0.001

Supplementary Figure 3:

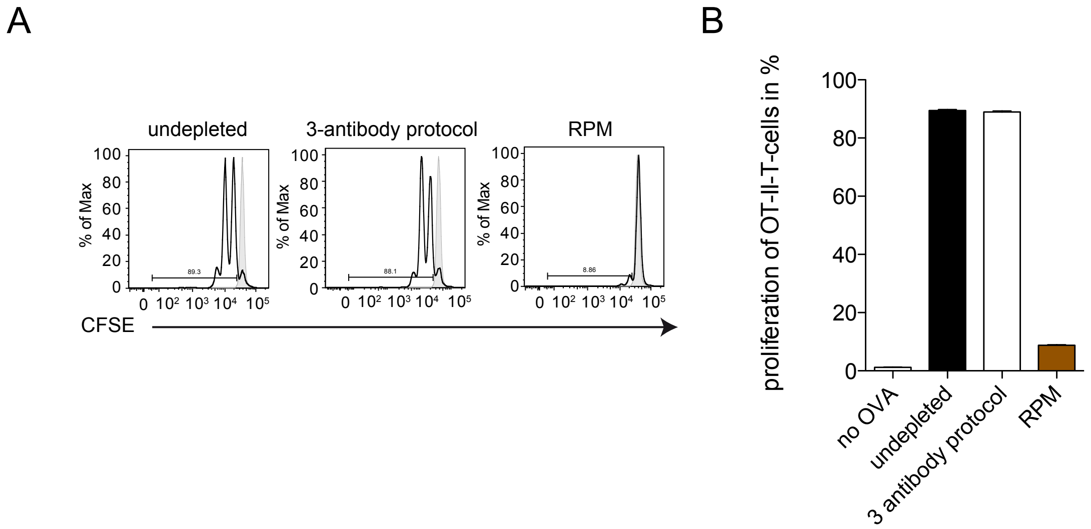

Supplementary Figure 3: Antigen presentation by CD11c^{+} cells and RPM obtained by MCS. The cells were purified by the standard protocol or depleted of RPM using our 3-antibody protocol. (A) Flow cytometrical analysis of the proliferation of CFSE-labeled OT-II T cells. Cells incubated with ovalbumine are depicted as black line, negative control that did not receive ovalbumin as grey background. (B) Statistical evaluation of (A).

Supplementary Figure 4:

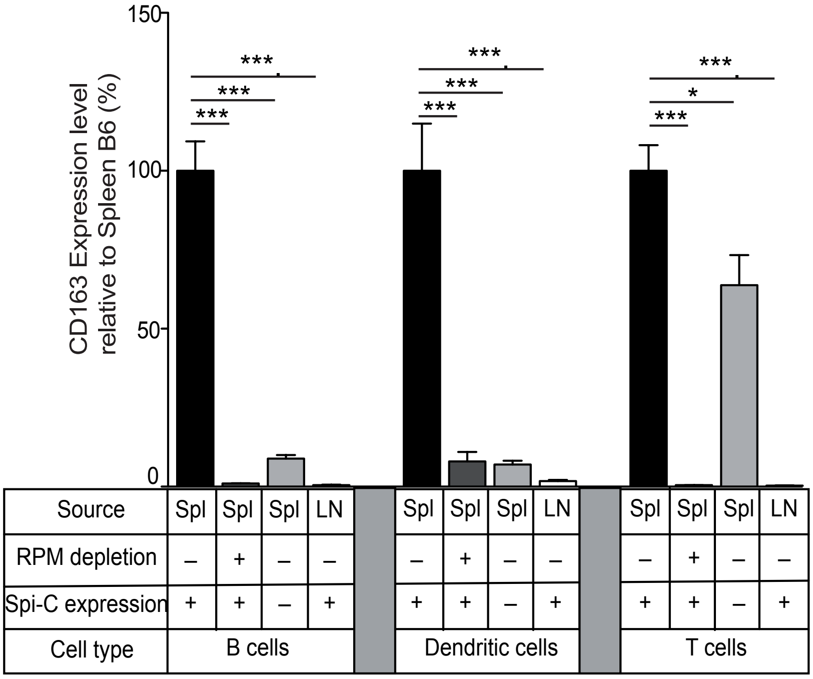

Supplementary Figure 4: RPM-contaminations are responsible for CD163 mRNA expression in splenic DCs, T cells or B cells isolated by conventional MCS. mRNA levels of CD163 determined by RT-PCR. Cell type, source tissue, SpiC-expression and RPM-depletion as indicated. Results are shown for one representative of two individual experiments (n=3); mean ± s.e.m.; *p< 0.05, ***p< 0.001 (One-way Anova in combination with a Bonferroni multiple-comparison test).

Supplementary Figure 5:

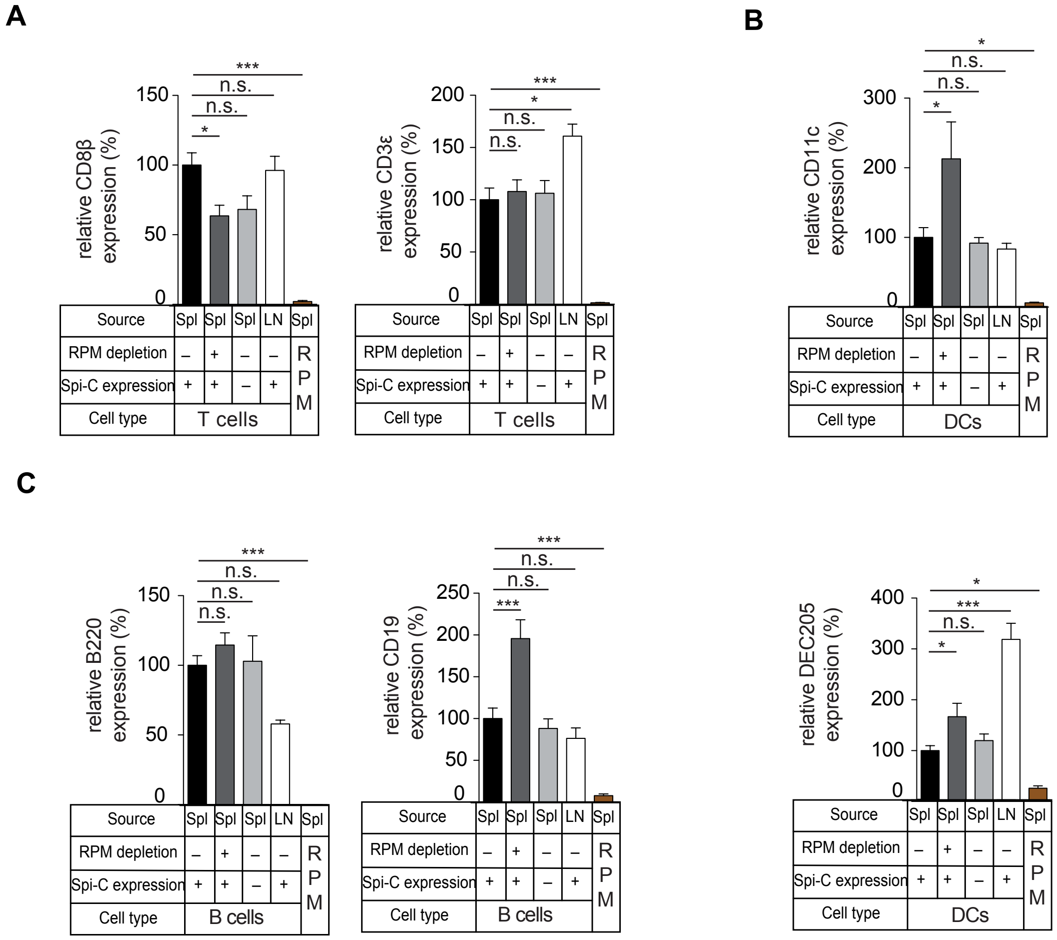

Supplementary Figure 5: RPM-depletion does not reduce the levels of non-RPM genes during RT-PCR analysis. (**A-C**) B cells, T cells and DCs were obtained by magnetic cell purification from spleens or the mesenteric lymph nodes without or with RPM-depletion using the 3-antibody protocol. The expression levels of the genes CD8β, CD3ε (**A**), CD11c, DEC205 (**B**), B220 and CD19 (**C**) relative to GAPDH was determined using qRT-PCR. As negative control expression of these genes was also determined in RPM purified by magnetic cell separation. Results are shown for one representative of two to three individual experiments using 2-4 mice per group. Error bars, s.d. (n=3 mice); *P< 0.05; ***P< 0.001

Supplementary Figure 6:

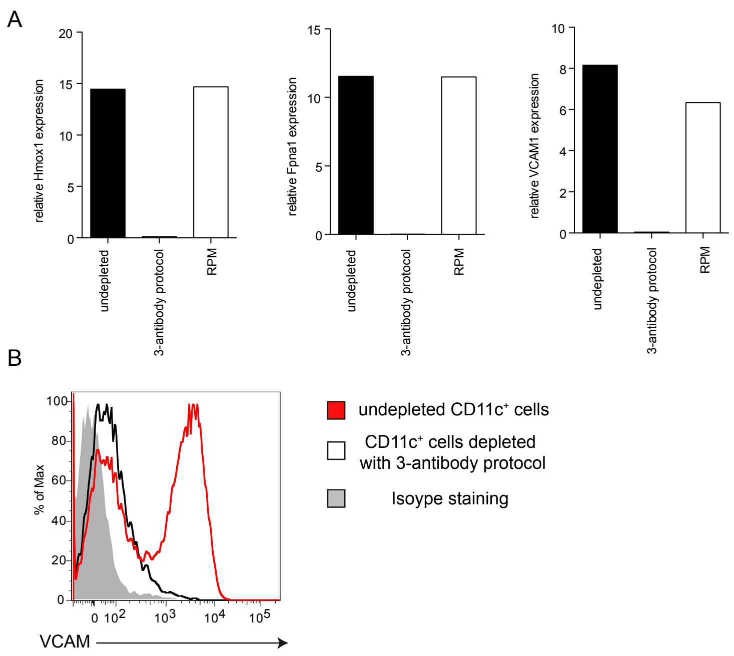

Supplementary figure 6: Genes involved in iron recycling can be detected in MCS purificates of CD11c$^+$ cells. (A) RT-PCR analysis of CD11c$^+$ cells and RPM obtained by MCS. The cells were purified by the standard protocol or depleted of RPM using our 3-antibody protocol. Depicted are the relative expression levels of Hmox1, Fpn1 and Vcam1. (B) Flow cytometric analysis of the VCAM1 expression in cells purified like in (A). CD11c$^+$ cells obtained by the standard MCS protocol are depicted as red line, CD11c$^+$ cells depleted of RPM using our 3-antibody protocol are depicted as black line. Isotype staining depicted as grey background.

Supplementary Figure 7:

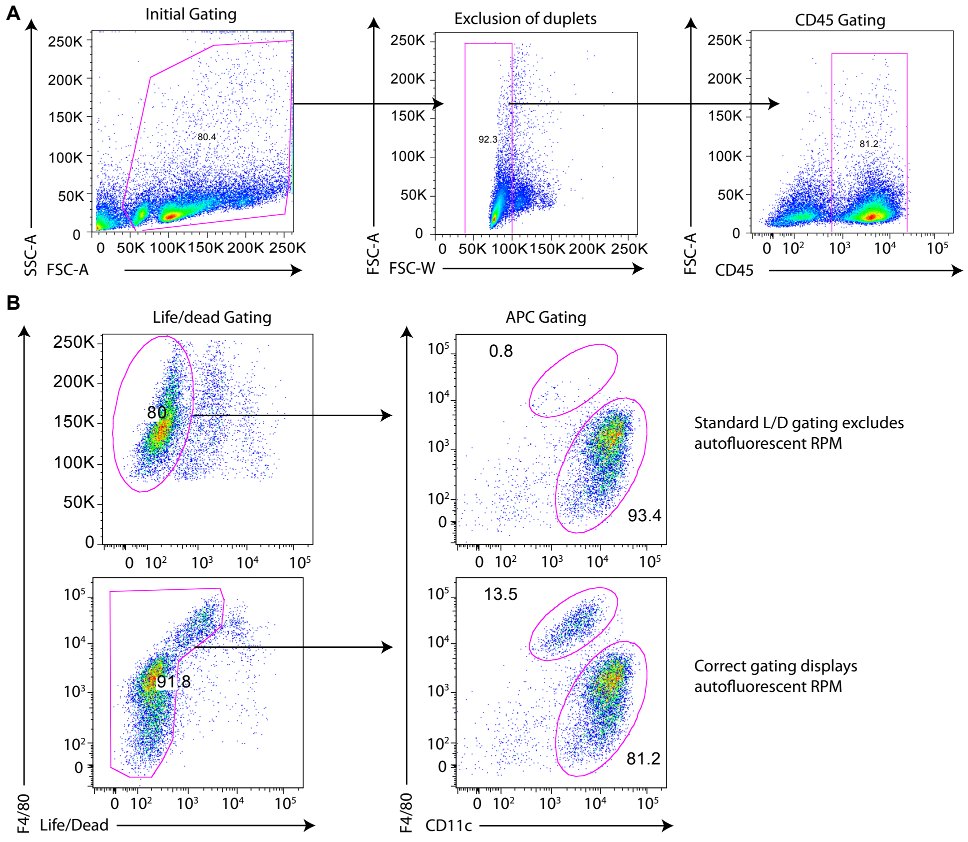

Supplementary Figure 7: Gating scheme employed for flow cytometrical analysis of splenocytes and MCS isolates. (A) Depicted is the initial gating to exclude small cells fragments (SSC-A versus FSC-C, left dot plot), cell duplets (FSC-A versus FSC-W, middle dot plot) and the gating on $CD45^+$ cells (FSC-A versus CD45, right dot plot). (B) Gating scheme for the exclusion of dead cells and RPM from flow cytometrical analysis. Dead cells are excluded by staining with a dead cell detection dye. RPM are either left in the viable cell population (upper dot plot) or are excluded as shown (lower dot plot). The dot plots on the right demonstrate the composition of the viable cell population.

Supplementary Table 1: Primers used for RT-PCR

Gene	Sense	Antisense
GITRL	CACTCAAGCCAACTGCCATC	CACAGGAATCACTTGGCCGT
PPARγ	TGTGAGACCAACAGCCTGAC	GTGTCAACCATGGTAATTTCAGT
B220	CACCTACACCCAGTGATGAACT	TCAGCTTGGCTGCTGAATGT
CD19	AAACCTGACCATCGAGAGGC	ACTTTGAAGAATCTCCTGGCGG
CD3ε	CCTCCTAGCTGTTGGCACTT	AGTAGCCACTGTCCTCGACT
CD8β	CTGCCCCAACCAAGAAGACT	TCTCCTCCGCACACAGTAAA
DEC205	GGCCTGTCTCAGCATTCACT	GTCCGGGCTCAGAGGAATTT
CD163	CACGGCACTCTTGGTTTGTG	GGAATTTTCCGAGGATTTCAGCA
CD11c	TTCCTGGCTGTTGGCTTGTG	GGAACTGATGCTACCCGAGC
MR	CGCTCGGACGGATGGCTCTG	GCTTGCAGCTTGCCCTTGCC
Hmox1	GCTTTAAGCTGGTGATGGCT	AGTGAGGCCCATACCAGAAG
Fpn1	CCCTGCTCTGGCTGTAAAAG	GGTGGGCTCTTGTTCACATT
Vcam1	ATTTTCTGGGGCAGGAAGTT	ACGTCAGAACAACCGAATCC
